# Antibacterial and Anti-Influenza Activities of *N*-Heterocyclic Carbene–Gold Complexes

**DOI:** 10.3390/ph17121680

**Published:** 2024-12-12

**Authors:** Michele Pellegrino, Paola Checconi, Jessica Ceramella, Carla Prezioso, Dolores Limongi, Maria Marra, Annaluisa Mariconda, Alessia Catalano, Marta De Angelis, Lucia Nencioni, Maria Stefania Sinicropi, Pasquale Longo, Stefano Aquaro

**Affiliations:** 1 Department of Pharmacy, Health and Nutritional Sciences, University of Calabria, Via Pietro Bucci, 87036 Arcavacata di Rende, Italy; michele.pellegrino@unical.it (M.P.); mariamarra1997@gmail.com (M.M.); s.sinicropi@unical.it (M.S.S.); 2 Department for the Promotion of Human Sciences and Quality of Life, San Raffaele University, Via di Val Cannuta 247, 00166 Rome, Italy; carla.prezioso@uniroma5.it (C.P.); dolores.limongi@uniroma5.it (D.L.); 3 Laboratory of Microbiology, IRCCS San Raffaele Roma, Via di Val Cannuta 247, 00166 Rome, Italy; 4 Department of Basic and Applied Sciences, University of Basilicata, Via dell’Ateneo Lucano 10, 85100 Potenza, Italy; annaluisa.mariconda@unibas.it; 5 Department of Pharmacy-Drug Sciences, University of Bari “Aldo Moro”, 70126 Bari, Italy; alessia.catalano@uniba.it; 6 Department of Public Health and Infectious Diseases, Laboratory Affiliated to Istituto Pasteur Italia-Fondazione Cenci Bolognetti, Sapienza University of Rome, 00185 Rome, Italy; marta.deangelis@uniroma1.it (M.D.A.); lucia.nencioni@uniroma1.it (L.N.); 7 Laboratory of Virology, Department of Molecular Medicine, Sapienza University, 00185 Rome, Italy; 8 Department of Chemistry and Biology “A. Zambelli”, University of Salerno, Via Giovanni Paolo II 132, 84084 Fisciano, Italy; plongo@unisa.it; 9 Department of Life, Health and Environmental Sciences, University of L’Aquila, Piazzale Salvatore Tommasi 1, Blocco 11, Coppito, 67010 L’Aquila, Italy; stefano.aquaro@univaq.it

**Keywords:** antimicrobials, influenza virus, drug resistance, *N*-heterocyclic carbene–gold complexes, metal-based therapeutics

## Abstract

Background/Objectives: Infectious diseases represent a serious threat due to rising antimicrobial resistance, particularly among multidrug-resistant bacteria and influenza viruses. Metal-based complexes, such as *N*-heterocyclic carbene–gold (NHC–gold) complexes, show promising therapeutic potential due to their ability to inhibit various pathogens. Methods: Eight NHC–gold complexes were synthesized and tested for antibacterial activity against *Escherichia coli*, *Enterococcus faecalis, Staphylococcus aureus*, and for anti-influenza activity in lung and bronchial epithelial cells infected with influenza virus A/H1N1. Antibacterial activity was assessed through the determination of the minimum inhibitory concentration (MIC) and the minimum bactericidal concentration (MBC), while the viral load was quantified using qRT-PCR. Results: Complexes 3, 4, and 6 showed significant antibacterial activity at concentrations of 10–20 µg/mL. Additionally, these complexes significantly reduced viral load, with complexes 3 and 4 markedly inhibiting replication. Conclusions: These findings support the potential use of NHC–gold complexes in combined antimicrobial and antiviral therapies, representing an attractive option for fighting resistant infections.

## 1. Introduction

Infectious diseases represent an important threat for humankind since they result in significant morbidity and are still among the leading causes of death worldwide [1]. The World Health Organization (WHO) has expressed serious concern regarding the continued increase in the development of multidrug resistance, especially among bacteria considered as “superbugs” [2]. Causes related to this growing phenomenon are found in the ability of pathogenic bacteria to transfer genetic materials conferring drug resistance as well as the inappropriate prescription and use of antimicrobial therapies, which provide resistant bacteria with a selective advantage [3]. Pathogenic viruses are not any easier, as the SARS-CoV-2 pandemic has shown us, and they are strictly interconnected with infectious disease. In fact, COVID-19 is exacerbating antimicrobial resistance [4]. A systematic review reported that, despite the fact that 6.9% of COVID-19 hospitalized patients were with bacterial co-infections, 72% received antibiotics, therefore, without a real clinical indication [4,5]. Among emerging pathogens, RNA viruses occupy a predominant place because of some of their characteristics: the rate of error during RNA replication and the consequent high potential for mutation; and the fact that most of them, such as corona- and influenza viruses, are zoonotic agents that can be transmitted, at least initially, from animals, such as birds and mammals, to humans [6]. In addition, due to the high mutation rate, there is also an increase in viral resistance to antiviral drugs, such as anti-influenza M2 inhibitors. It is for this reason that they are no longer recommended [7]. This increase in both antibacterial and antiviral drug resistance has not been paralleled by the development of new antimicrobials, even if different approaches and strategies are the object of studies, including drug repositioning and/or the search for new compounds that can be directly administered to host targets [8,9,10]. Moreover, a few antimicrobials have useful activity across the different pathogenic groups.

The biological activities of metal-based complexes have been of great interest for a long time due to their multiple mechanisms of action and broad spectrum of effects, such as antitumor, antioxidant, and antimicrobial [11]. Particularly, integrating gold ions into organic molecules allows for more efficient transport and targeted release, and, as a result, gold-based drugs are playing an increasingly significant role in drug discovery and medicinal chemistry [12]. Moreover, the enhanced stability and tunable lipophilicity of gold *N*-heterocyclic carbene (NHC) complexes are key factors in improving their biological properties [13]. The attractive and remarkable biological properties possessed by gold–NHC complexes that include, for instance, antibiotic, anticancer, antioxidant, and anti-inflammatory ones are also accompanied by very low side effects [13]. The application of gold complexes as innovative antibiotics, against *Mycobacterium tuberculosis*, was reported by R. Koch (Robert Koch, in “Verhandlungen des X. Internationalen Medizinischen Kongresses”) [14], but, more recently, these features have been investigated and strengthened [15,16]. Gold complexes bearing *N*-heterocyclic carbene ligands or phosphines are the most common complexes that were also tested as antivirals [17,18,19,20].

In this study, antimicrobial activity, i.e., the antibacterial and antiviral, particularly anti-influenza activity, of eight NHC–gold complexes, indicated as 1–8, were investigated. We found that gold-based complexes 3, 4, and 6 showed significant antibacterial activity; in addition, complexes 3 and 4 markedly inhibited influenza virus replication.

## 2. Results

### 2.1. Synthesis of the NHC-Au Complexes

Gold complexes 1–8 were prepared using procedures reported in the literature by some of us: 1, 3 [21], 2, 4, 6 and 8 [22], 5 [23], and 7 [24]. The characterization results were consistent with the data previously reported.

**Figure 1:**
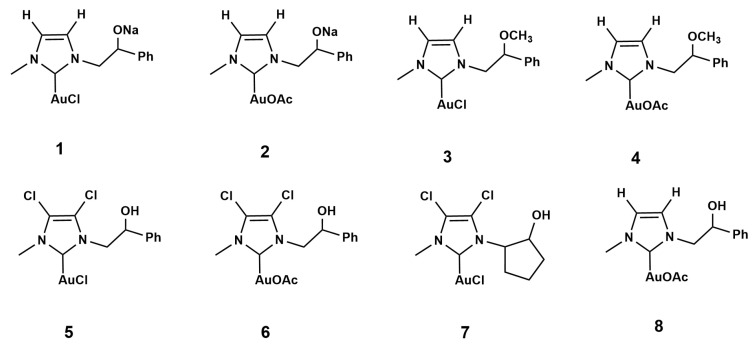
*N*-heterocyclic carbene–gold complexes (1–8).

All Au(I) complexes depicted in Figure 1 are stabilized by a neutral ligand based on an *N*-heterocyclic carbene (NHC), which is asymmetrically substituted at the nitrogen atoms of the ring and by a charged ligand. The nature of the counterions varies between the different complexes, i.e., a chloride anion for four of them and an acetate anion for the other four complexes. In addition, there is a further additional structural difference between the imidazole rings. In particular, the carbons in the 4 and 5 positions of the ring can be bonded to hydrogen or chlorine atoms. This, obviously, influences the electronic properties and, hence, the reactivity of the complexes.

### 2.2. Antibacterial Activity

All the synthesized gold complexes 1–8, shown in Figure 1, were investigated for antibacterial activity against both the gram (+)/(−) bacteria. The DMSO did not exhibit any antimicrobial activity; conversely, all strains used resulted in ampicillin-sensible properties. The antimicrobial activity, in terms of the minimum inhibitory concentration (MIC) and minimum bactericidal concentration (MBC), expressed as µg/mL, is shown in Table 1. Our data evidenced that the gold complexes showed a significant antibacterial activity in all strains tested.

Particularly, as shown in Table 1, the complexes 3, 4, and 6 are able to inhibit the strain-tested growth at a 10 or 20 µg/mL concentration. Conversely, for the other tested complexes, a greater concentration (from 50 to 75 µg/mL) is required to obtain the MIC values.

Overall, these data indicate that the synthesized complexes represent very promising antibacterial candidates for use either alone or synergistically in the treatment of multifactorial/multisymptomatic diseases.

### 2.3. Antiviral Activity

**Figure 2:**
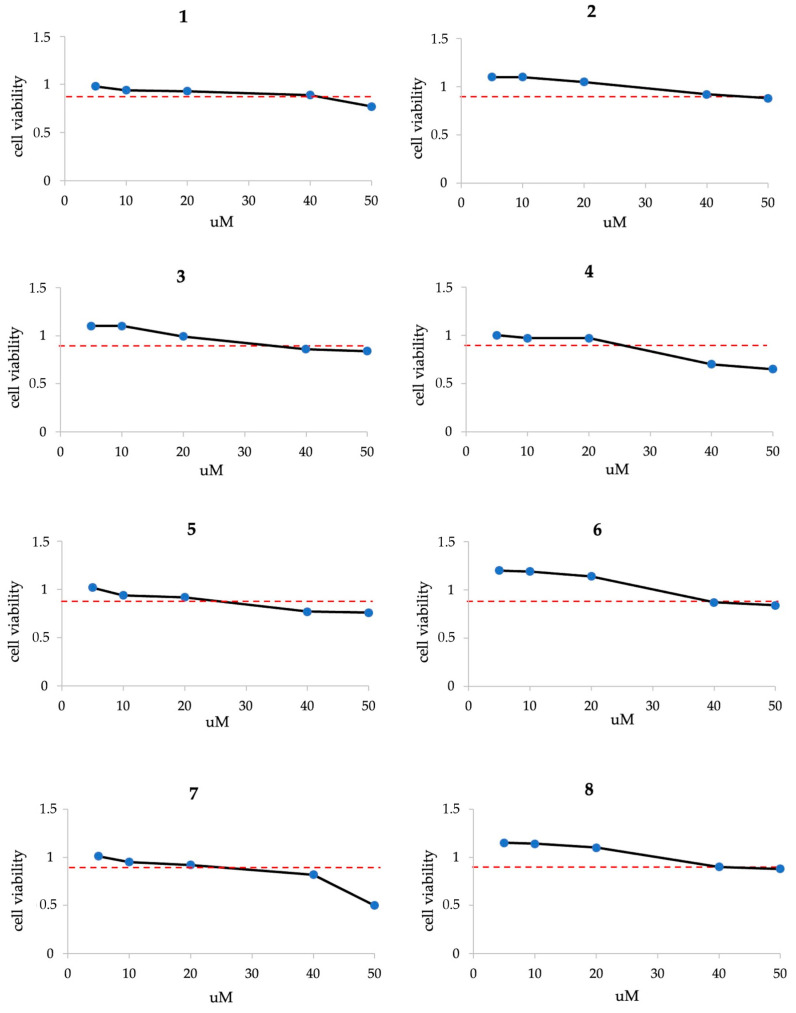
Cell viability (ratio to control) of A549 cells treated with different concentrations of complexes 1–8 (5, 10, 20, 40, and 50 μM, blue dots) for 24 h evaluated by MTT assay. The red dotted line indicates a ratio of 0.9.

**Figure 3:**
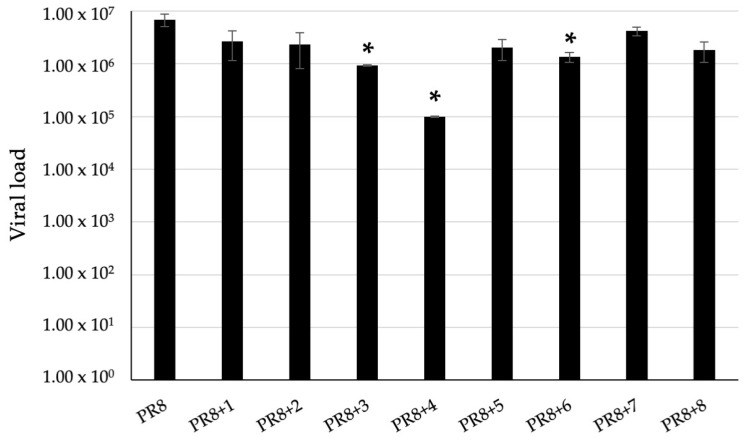
Viral load quantified in supernatants from PR8-infected A549 cells, treated with complexes 1–8 (20 μM) for 24 h, by qRT-PCR. * *p* < 0.01.

In order to study a potential antiviral, in particular, the anti-influenza virus activity of the gold complexes, we preliminarily evaluated its effect on the pulmonary cell line A549 viability. Cells were treated with different concentrations of the molecules, ranging from 5 to 50 μM, for 24 h, and cell viability was evaluated by the 3-(4,5-dimethyl-2-thiazolyl)-2,5 diphenyl tetrazolium bromide (MTT) assay, as described in the Materials and Methods Section. The values of cell viability were higher than 0.9 (the ratio to control cells) until the concentration of 20 μM (Figure 2), which then was chosen for the following experiments:

To evaluate the antiviral activity, A549 cells were infected with the Influenza A/Puerto Rico/8/34 H1N1 (PR8) virus and treated with each compound (20 μM) for 24 h of infection. The viral load was quantified in the supernatants by quantitative reverse transcription-PCR (qRT-PCR, see Section 4). The supernatants from 3, 4, and 6-treated, infected cells showed a viral load significantly lower (*p* < 0.01) compared to those from infected cells (Figure 3).

**Figure 4:**
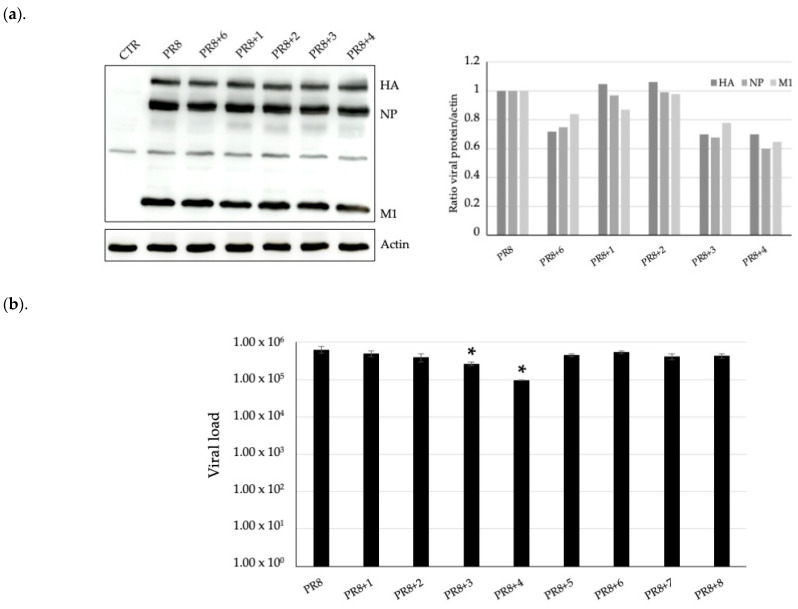
Western blot analysis, with anti-influenza antibody, of influenza virus proteins in cell lysates (**a**) and the viral load measured in supernatants by qRT-PCR (**b**) from A549 cells infected with supernatants from the previous experiments, * *p* < 0.01. In (**a**), actin was used as the loading control, and densitometric analysis was shown in the graph on the right of the Western blot image, expressed as the ratio of each viral protein (HA, NP, M1) to actin.

To evaluate the infectivity of supernatants of the same samples, they were used in a second set of experiments to infect A549 cells; after 24 h of infection, cell lysates were analyzed for viral proteins by immunoblotting. As shown in Figure 4a, the viral protein expression was lower. When cells were treated with complexes 1–4 and 6 to a different extent, the following occurred: densitometric analysis (Figure 4a, on the right) revealed a reduction for all the three main PR8 virus proteins, hemagglutinin (HA), nucleoprotein (NP), and matrix protein1 (M1), with complexes 3–4 and 6; and a major reduction was observable for complex 4. Protein expression did not vary with the other compounds. Viral load was measured again in the supernatants. The results confirmed that complexes 3 and 4 were able to significantly inhibit viral replication (Figure 4b).

**Figure 5:**
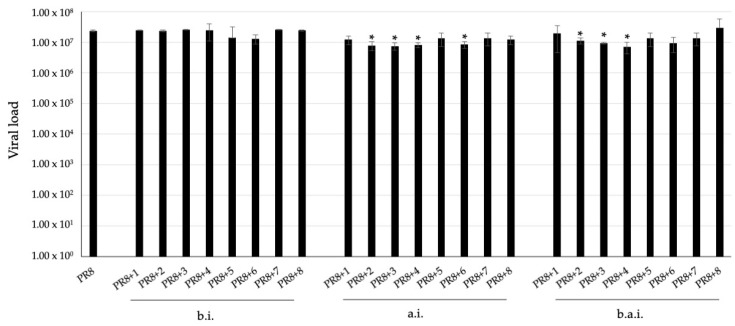
Viral load quantified by qRT-PCR in supernatants from PR8-infected BEAS-2B cells treated with complexes 1–8 (20 μM) 1h before infection (b.i.), after infection (a.i.), and both 1h before and after infection, for 24 h. * *p* < 0.05.

To confirm the antiviral activity of the gold complexes, a different and more suitable cell culture model for influenza virus, i.e., non-tumorigenic bronchial epithelial cells, BEAS-2B, was used. Ruled out for the complexes’ cytotoxicity, these cells were treated with the compounds at 20 μM at different times from PR8 infection: 1 h before infection, after infection, and both 1h before and after infection, for the following 24 h. As shown in Figure 5, the treatment before infection had no significant effect. Instead, the treatment after infection confirmed that complexes 3, 4, 6, and, in this model, the complex 2, are able to significantly reduce viral load. Finally, the treatment both 1h before and after infection had similar results to the treatment after infection.

## 3. Discussion

To face the challenges of emerging pathogens and multidrug-resistance phenomena, different approaches and strategies are under investigation, including drug repositioning and the search for new compounds that can be directly administered to microbial targets and/or host targets [26,27,28,29]. Metal-based complexes, including gold-based ones, have long garnered great interest due to their multiple mechanisms of action and spectrum of effects [30,31]. On the other hand, NHC complexes also represent an important class of agents with broad biological properties [32,33,34].

In the present study, the antibacterial and antiviral activity of eight NHC–gold complexes were investigated. We found that all the complexes showed an antibacterial activity against all strains tested (one Gram-negative bacterial strain, *Escherichia coli*, and two Gram-positive bacterial strains, *Enterococcus faecalis* and *Staphylococcus aureus*), with the complexes 3, 4, and 6 able to inhibit the strains at a 10 or 20 µg/mL concentration; meanwhile, for the other tested complexes, a greater concentration (from 50 to 75 µg/mL) is required to obtain the MIC values. The lowest concentration capable of inhibiting the strains tested is in line with previously reported studies that highlight the efficacy of gold complexes as antimicrobial agents against a broad spectrum of pathogens, including *Escherichia coli* and *Staphylococcus aureus* [35,36,37]. The ability of gold complexes to inhibit microbial growth is largely attributed to their interaction with critical bacterial enzymes, such as thioredoxin reductase (TrxR), which plays a pivotal role in maintaining redox homeostasis within bacterial cells. Inhibiting TrxR disrupts the bacterial defense mechanism against oxidative stress, leading to cellular damage and death [38,39]. Other mechanisms for the antimicrobial activity could involve the efficient interactions with different targets, including the potential inhibition of actin polymerization, which disrupts cellular structure and essential functions in microbial cells [32,40].

In terms of antiviral activity, the gold complexes 3, 4, and 6 significantly reduced the replication of the influenza A virus in an in vitro infection model using lung epithelial cells. This reduction in viral load and infectivity without inducing cytotoxicity on host cells demonstrates the potential of these compounds as antiviral agents. Notably, compounds 3 and 4 significantly inhibited viral replication, reducing infectious viral particle production, as shown in the second round of infection, i.e., infection by supernatants from the first infection experiments (Figure 4). The efficacy in reducing viral load by gold complexes 3, 4, and 6 was confirmed in another cell culture model represented by BEAS-2B, non-tumorigenic bronchial epithelial cells. Moreover, viral load was reduced by complex 2 in this model, suggesting that the mechanisms of action could involve both viral and cell factors. In addition, the treatment at different times from infection, specifically before, after, and both before and after infection, resulted in a viral load reduction only when performed after infection, indicating that complexes act after viral adsorption and entry (Figure 5).

The efficacy of different gold complexes as antivirals were also reported by other groups against different families of viruses, including retrovirus [41], flavivirus [42], and coronaviruses [19,20,43]. Regarding the mechanisms of action, a significant interaction of some gold compounds with dsRNA for Chikungunya virus has been shown, suggesting that they could inhibit viral replication by dsRNA binding [42]; some other gold drugs have been shown to inhibit the papain-like protease (PLpro) of SARS-CoV-1 and SARS-CoV-2, which is a key enzyme in the viral replication of these coronaviruses. The activity of the complexes against both PL proenzymes correlated with the ability of the inhibitors to remove zinc ions from the zinc center of the enzyme. Moreover, they were effective inhibitors of the interaction of the SARS-CoV-2 spike protein with the angiotensin-converting enzyme 2 (ACE2) host receptor and, thus, might interfere with the viral entry process [43]. However, the latter mechanism would not seem to be involved in our study, as the NHC–gold complexes act in a post-entry phase.

Indeed, further studies are warranted to elucidate the exact molecular interactions of the studied complexes with viral RNA, proteins, and bacterial targets.

Moreover, the broad-spectrum antimicrobial activity observed in this study suggests that these complexes may act on host molecular pathways, activated from different pathogens as redox-modulated pathways, which could inform the development of new therapeutic strategies against influenza and/or other viral and bacterial pathogens.

Our findings also underscore the potential of these compounds for use in combination therapies.

Future work should explore the synergistic effects of these compounds with existing antiviral and antibacterial agents to maximize therapeutic outcomes.

## 4. Materials and Methods

### 4.1. Synthesis of NHC–Gold Complexes 1–8

All the chemicals and reagents were purchased from Merck Serono Spa and TCI Chemicals. The NMR spectra were recorded on a Bruker AM 300 and Bruker AVANCE 400 spectrometer (300 and 400 MHz for ^1^H; 75 and 100 MHz for ^13^C, respectively). NMR samples were prepared by dissolving about 10 mg of compounds in 0.5 mL of deuterated solvent. All chemical shifts are reported in ppm using the residual proton impurities of the deuterated solvents. ^1^H-NMR were reported relative to CD_2_Cl_2_ δ 5.32 ppm and DMSO-*d_6_* δ 2.50 ppm; ^13^C-NMR were reported relative to CD_2_Cl_2_ δ 54.00 ppm and DMSO-*d_6_* δ 39.52 ppm. Multiplicities are abbreviated as follows: singlet (s), doublet (d), multiplet (m), and broad (br). ESI-MS measurements of organic compounds were performed on a Waters Quattro Micro triple quadrupole mass spectrometer equipped with an electrospray ion source. MALDI-MS mass spectra were acquired using a Bruker SolariX XR Fourier transform ion cyclotron resonance mass spectrometer (Bruker Daltonik GmbH, Bremen, Germany) equipped with a 7 T refrigerated, actively shielded superconducting magnet (Bruker Biospin, Wissembourg, France). The samples were ionized in positive ion mode using the MALDI ion source (Bruker Daltonik GmbH, Bremen, Germany). The mass range was set to *m*/*z* 200–3000. The laser power was 28%, and 22 laser shots were used for each scan. The mass spectra were calibrated externally using a mix of peptide clusters in MALDI ionization positive ion mode.

The gold complexes 1–8 were prepared using procedures reported in the literature: 1,3 [21], 2, 4, 6 and 8 [22], 5 [23], and 7 [24].

*Complex 1 (bis-[N-methyl,N′-[(2-sodium alcoholate-2-phenyl)ethyl])-imidazole-2-ylidine]gold(I)]^+^[dichloride-gold]^−^*

Yield: 59%. ^1^H NMR (CD_2_Cl_2_, 300 MHz, ppm): *δ* 7.36 (m, 5H, aromatic protons), 7.01 (s, 1H, NCHCHN), 6.95 (s, 1H, NCHCHN), 5.21 (t, 1H, CHO^−^, *J_anti_* = 7.22 Hz, *J_gauche_* = 5.47 Hz), 4.12 (d, 2H, NCH*_2_*, *J_anti_* = 7.27 Hz, *J_gauche_* =5.47 Hz, *J_gem_*, 1.26 Hz), and 3.80 (s, 3H, NCH_3_). ^13^C {^1^H} NMR (CD_2_Cl_2_, 100 MHz, ppm): *δ* 171.9 (NCN), 141.7 (ipso carbon aromatic ring), 129.7, 129.5, 129.0 (aromatic carbons), 123.0, 121.9 (NCHCHN), 74.2 (CHO^−^), 58.8 (NCH_2_), and 38.5 (NCH_3_). ESI *m*/*z*: 612.5 Da attributable to [C_22_H_18_AuN_4_O_2_Na_2_]^+^.

*Complex 2 bis-[(N-methyl, N′-[(2-sodium alcoholate-2-phenyl-ethyl)imidazole-2-ylidine]gold(I)] acetate*

Yield: 42%. ^1^H NMR (400 MHz, CDCl_3_, ppm): *δ* 7.34 (m, 5H, aromatic protons), 6.88 (d, 1H, NCHCHN), 6.85 (d, NCHCHN), 5.22 (m, 1H, CHO^−^), 4.44–4.39 (m, 2H, NCH_2_), 3.77 (s, 3H, NCH_3_), 1.98 (s, 3H, OCOCH_3_). ^13^C NMR (75 MHz, CDCl_3_, ppm): *δ* 176.0 (CH_3_COO), 171.2 (NCN), 141.9 (ipso carbon aromatic ring), 128.6, 126.3, 126.1 (aromatic carbons), 123.2, 121.2 (N*C*HCHN), 72.8 (CHO^−^), 58.5 (NCH_2_), 38.1 (N*C*H_3_), and 22.5 (CH_3_COO). ESI m/z of 612.7 Dalton attributable to [C_22_H_17_AuN_4_O_2_Na_2_]^+^.

*Complex 3 bis-[N-methyl-N′(2-metoxy-2-phenyl)ethyl-imidazole-2-ylidene gold(I)]^+^ [dichloride-gold]^−^*

Yield: 59%. ^1^H NMR (400 MHz, DMSO-*d_6_*, ppm): *δ* 7.32 (m, 7H, aromatic protons), 4.65 (m, 1H, CHOCH_3_ *J_anti_* = 7.77 Hz, *J_gauche_* = 6.28 Hz), 4.22 (dd, 2H, NCH_2_CHOCH_3_ *J_anti_* = 7.74 Hz, *J_gauche_* = 6.28 Hz, *J_gem_* = 1.83 Hz), 3.74 (s, 3H, NCH_3_), and 3.00 (s, 3H, CHOCH_3_). ^13^C NMR (100 MHz, DMSO-*d_6_*, ppm): *δ* 169.3 (NCN), 137.9 (ipso carbon aromatic ring), 128.7, 128.4, 126.7 (aromatic carbons), 122.4, 122.2 (NCH*C*HN), 82.4 (CHOCH_3_), 56.7 (NCH_2_), 55.9 (OCH_3_), and 37.3 (NCH_3_). (MALDI, CH_2_Cl_2_): *m*/*z* = 629.20 attributable to [(C_26_H_32_N_4_O_2_)Au]^+^.

*Complex 4 bis-[(N-methyl, N′(2-methoxy-2-phenyl-ethyl)imidazole-2-ylidine]gold(I)] acetate*

Yield: 55%. ^1^H NMR (300 MHz, DMSO-*d_6_*, ppm): *δ* 7.37 (m, 7H, Ph ring + NCHCHN), 4.765 (m, 1H, CHOCH_3_), 4.22 (m, 2H, NCH_2_), 3.71 (s, 3H, NCH_3_), 3.09 (s, 3H, OCH_3_), and 1.75 (s, 3H, OCOCH_3_). ^13^C NMR (75 MHz, DMSO-*d_6_*, ppm): *δ* 174.8 (O*C*OCH_3_), 162.6 (N*C*N), 138.1 (ipso carbon of aromatic ring), 128.5, 128.4, 126.9 (aromatic carbons), 122.7, 122.2 (NCHCHN), 82.1 (CHOCH_3_), 56.6 (OCH_3_), 55.8 (NCH_2_) 37.5 (NCH_3_), and 24.3 (CH_3_COO). ESI = *m*/*z* 629.22 Dalton attributable to [C_26_H_32_AuN_4_O_2_]^+^.

*Complex 5 bis-[4,5-dichloro-(N-methy-N′(2-hydroxy-2-phenyl)ethyl-imidazole-2-ylidene]gold(I)]^+^[dichloro-gold]^−^*

Yield: 47%. ^1^H NMR (400 MHz, DMSO-*d_6_*, ppm): *δ* 7.31 (m, 5H, aromatic protons), 5.89 (br, 1H, OH), 5.11 (m, 1H, CHOH), 4.243 (m, 2H, NCH_2_CHOH), and 3.80 (s, 3H, CH_3_). ^13^C NMR (100 MHz, DMSO-*d_6_*, ppm): *δ* 170.9 (N*C*N), 141.1 (ipso carbon of aromatic ring), 128.2, 127.8, 127.2, 125.8 (aromatic carbons), 117.1, 116.5 (NCClCClN), 72.1 (CH_2_CHOH), 56.6 (NCH_2_), and 37.0 (NCH_3_). MALDI, CH_2_Cl_2_: *m*/*z* = 739.03 Da attributable to [C_24_H_24_N_4_O_2_Cl_4_]Au^+^.

*Complex 6 bis-[4,5-dichloro-(N-methyl, N’(2-hydroxy-2-phenyl-ethyl)imidazole-2-ylidine]gold(I)] acetate*

Yield: 55%. ^1^H NMR (400 MHz, DMSO-*d_6_*, ppm): *δ* 7.41 (m, 5H, aromatic protons), 5.82 (br, 1H, OH), 5.21 (m, 1H, CHOH), 4.20 (m, 2H, NCH_2_), 3.86 (s, 3H, NCH_3_), and 1.81 (s, 3H, COCH_3_). ^13^C NMR (75 MHz, DMSO-*d_6_*, ppm): *δ* 174.9 (OCOCH_3_), 163.7 (NCN), 141.6 (ipso carbon of aromatic ring), 128.3, 127.4, 125.7 (aromatic carbons), 117.6, 116.5 (NCClCClN), 72.6 (CHOH), 56.2 (NCH_2_) 37.9 (NCH_3_), and 23.8 (CH_3_COO). MALDI, CH_2_Cl_2_: *m*/*z* = 739.07 Dalton attributable to [C_24_H_24_AuCl_4_N_4_O_2_]^+^.

*Complex 7 bis-[N-methyl,N′(cyclopentane-2ol)-imidazole-2-ylidine]gold(I)]^+^[di-chlorogold]^−^*

Yield: 55%. ^1^H NMR (400 MHz, CD_2_Cl_2_, ppm): *δ* 6.98 (s, 1H, NCHCH), 7.05 (s, 1H, NCHCH), 4.88 (m, 1H, OCH), 4.49 (m, 1H, NCH), 3.82 (s, 3H, NCH_3_), 2.56 (m, 2H, OCHCH_2_), 2.22 (m, 2H, NCHCH_2_), and 1.79 (m, 2H, CH_2_CH_2_CH_2_). ^13^C NMR (100 MHz, CD_2_Cl_2_, ppm): *δ* 169.8 (NCN), 124.8, 122.5 (N*C*HCHN,) 78.3 (O*C*H), 69.8 (N*C*H), 45.9 (NCH_3_), 39.6 (OCHCH_2_), 34.1 (NCHCH_2_), and 28.1 (CH_2_CH_2_CH_2_). ESI = *m*/*z* 531.19 Dalton attributable to [C_18_H_29_AuN_4_O_2_]^+^.

*Complex 8 bis-[N-methyl, N′(2hydroxy-2-phenyl-ethyl)imidazole-2-ylidine]gold(I)] acetate*

Yield: 54%. ^1^H NMR (400 MHz, DMSO-*d_6_*, ppm): *δ* 7.38 (m, 7H, Ph ring + NCHCHN), 5.13 (m, 1H, CHOH), 4.11 (m, 2H, NCH_2_), 3.73 (s, 3H, NCH_3_), and 1.86 (s, 3H, OCOCH_3_). ^13^C NMR (100 MHz, DMSO-*d_6_*, ppm): *δ* 174.6 (OCOCH_3_), 161.8 (N*C*N), 142.2 (ipso carbon of aromatic ring), 128.1, 127.7, 126.1 (aromatic carbons), 122.5, 122.2 (NCHCHN), 72.1 (CHOH), 57.9 (NCH_2_) 37.1 (NCH_3_), and 24.5 (CH_3_COO). MALDI-MS = *m*/*z* 601.19 Dalton attributable to [C_24_H_28_AuN_4_O_2_]^+^.

### 4.2. Minimum Inhibitory Concentration (MIC) and Minimum Bactericidal Concentration (MBC) Determination

One Gram-negative bacterial strain [*Escherichia coli* (ATCC^®^ 25922TM)], two Gram-positive bacterial strains [*Enterococcus faecalis* (ATCC^®^ 19433TM) and *Staphylococcus aureus* (ATCC^®^ 23235TM)] were used in the antibacterial tests to measure the MIC and MBC values. The MIC and MBC of the compounds tested were determined according to CLSI guidelines [44].

The MIC is interpreted as the lowest concentration that inhibits the compound’s visible microbial growth and is expressed in terms of μg/mL, whereas the MBC is interpreted as the lowest concentration that can completely kill the bacteria; both are interpreted using the broth dilution method [29]. Bacteria were grown overnight in a Mueller Hinton medium (MH 2%), diluted at a density of 4000 colony-forming units (CFUs/)mL, plated in a sterile 96-well microtiter to obtain a total inoculum load of ca. 105 cells/ well, and then treated with increasing concentrations of molecules (1, 5, 10, 20, 50, 75, 100, 125, 150 μg/mL). Successively, after the incubation plate was set at 37 °C for 18 h (overnight), the bacterial growth was monitored at a wavelength of 600 nm using a Multiskan spectrophotometer (Multiskan Ex Microplate model; Thermo Scientific, Nyon, Switzerland). MIC or MBC values were obtained by comparing the cell density with a positive control (bacterial cells grown in the MH medium were added with only the vehicle, DMSO). For each experiment, carried out five times, triplicate assays were performed. Finally, to verify the strain sensibility, an Ampicillin (Sigma Aldrich A9393, Darmstadt, Germany) standard antibiotic was used as the control [44].

### 4.3. Cell Culture and Complex Treatment

A549 human lung adenocarcinoma cells and BEAS-2B human bronchial epithelial cells were grown in the DMEM medium, supplemented with 10% fetal bovine serum (FBS), 0.3 mg/mL of glutamine, 100 U/mL of penicillin, and 100 μg/mL of streptomycin.

Au complexes were dissolved in DMSO and then diluted to the final concentration in the cell-culture medium. Cytotoxicity was evaluated on A549 cells by treating the cells with concentrations from 5 μM to 50 μM. After 24 h, the cytotoxicity of the treatments was evaluated by the 3-(4,5-dimethyl-2-thiazolyl)-2,5 diphenyl tetrazolium bromide (MTT) assay [45] and expressed as the reduction in viability of treated cells (ratio to control, i.e., cells treated with DMSO alone). For the evaluation of antiviral activity, complexes were diluted to the final concentration of 20 μM in the cell-culture medium and were added 1 h before virus infection, after infection, or both 1h before and after infection, for the 24 h of infection.

### 4.4. Virus Infection and Titration

Influenza virus A/Puerto Rico/8/34 H1N1 (PR8) was grown in the allantoic cavities of 10-day-old embryonated chicken eggs and harvested after 48 h at 37 °C. To perform a multi-cycle of infection, epithelial cells were challenged with PR8 at a multiplicity of infection (m.o.i.) of 0.1 for 1h at 37 °C, washed with PBS, and incubated with the medium supplemented with 2% FBS for 24 h. The virus titer was determined in the supernatants of infected cells by qRT-PCR, as described below.

### 4.5. Quantitative Reverse Transcription-PCR (qRT-PCR)

Reverse-transcription and quantitative PCR were performed using the AMPLilabTM Real-Time PCR System (Adaltis) with the following thermoprofile: 45 °C for 10 min and 95 °C for 5 min, followed by 45 cycles of 95 °C for 10 s and 64 °C for 30 s and 72 °C 10 s, with signal acquisition in the FAM channel at the end of the 64 °C annealing step. The qRT-PCR was carried out with Invitrogen SuperScript III Platinum One-Step quantitative RT-PCR kits (Life Technologies, Carlsbad, CA, USA) according to the manufacturer’s instructions. The Cq (quantification cycle) values were estimated by using plasmids containing a known copy number of amplification targets. The viral load (VL) was determined by comparing the quantification cycle (Cq) of the M gene amplification of the target sample with a standard curve obtained by plotting six standards ranging from 101 to 106 copies/reaction in triplicates. A threshold of 0.1 was defined for all reactions. The raw results are presented in copies/reaction (quantity) and were converted into copies/mL by the formula: [quantity/Rt-qPCR reaction volume] × [sample elution volume (μL)/sample input to extraction (mL)]. All results of VL quantification were presented in Log10 copies/mL [46].

### 4.6. Western Blot Analysis

Cells were lysed with a lysis buffer supplemented with phenylmethylsulfonyl fluoride (PMSF) and a protease inhibitor mixture (Sigma-Aldrich, Darmstadt, Germany) for 30 min on ice. Protein concentration was determined with DC Protein Assay (Bio-Rad, Hercules, CA, USA). Then, cell lysates were analyzed by SDS-PAGE under reducing conditions (by treatment with DTT) followed by Western blot. The proteins were visualized using the following primary and secondary (horseradish peroxidase-conjugated) antibodies: anti-Influenza (Merck Millipore, Darmstadt, Germany), anti-Actin (Sigma Aldrich); anti-goat, and anti-mouse (Bethyl Laboratories, Montgomery, TX, USA). Blots were developed with the WesternBright ECL HRP substrate (Advansta, San Jose, CA, USA). Densitometry analysis was performed using ImageJ version 1.54.

### 4.7. Statistical Analysis

Experiments were carried out at least three times, each performed in duplicate.

Statistical analysis was performed using a two-tailed Student’s test. A *p* value < 0.05 was considered statistically significant.

## 5. Conclusions

In conclusion, the synthesized NHC–gold complexes show significant promise as broad-spectrum antimicrobial agents, with the potential for applications in both antibacterial and antiviral therapies.

The higher biological activity observed for complexes **3** and **4** could be related to the presence of the methoxy group. This functional group makes the complexes more lipophilic than their congeners, potentially enhancing their ability to interact with lipid-rich environments, thereby increasing their overall biological effectiveness. The methoxy group influences the solubility and the ability to cross the cell membrane of these complexes, making them bioavailable and probably more capable of playing a crucial role in the drug delivery system. Their low effective concentration and ability to inhibit pathogen growth without inducing cytotoxicity make them ideal candidates for further preclinical development. Given the rise in antimicrobial resistance and the ongoing threat of viral pandemics, these gold complexes represent a valuable addition to the arsenal of antimicrobial agents.

## Figures and Tables

**Table 1:** MIC and MBC values of Au complexes.

Microorganisms						
Samples	*E. coli* ^[c]^		*S. aureus* ^[c]^		*E. faecalis* ^[c]^	
	MIC ^[a]^	MBC ^[b]^	MIC ^[a]^	MBC ^[b]^	MIC ^[a]^	MBC ^[b]^
1	20	100	20	100	50	150
2	20	100	50	150	50	150
3	20	100	10	100	20	100
4	20	100	10	100	10	100
5	75	150	75	150	75	150
6	20	100	10	100	10	100
7	75	150	50	150	50	150
8	20	100	50	150	50	150

MIC and MBC: µg/mL ^[a]^ minimum inhibitory concentration; ^[b]^ minimum bactericidal concentration; ^[c]^ and ampicillin-sensitive [25].

## Data Availability

Data are contained within this article.
